# Supplementary material for: Dynamic transcriptomic profiles of zebrafish gills in response to zinc supplementation
Source: BMC Genomics. 2010 Oct 11;11:553. doi: 10.1186/1471-2164-11-553 (PMC3091702; doi:10.1186/1471-2164-11-553)
Supplement: Additional file 2 — Interactive Direct Interaction Network representing the molecular interactions between zinc, copper, iron, calcium and proteins encoded by transcripts changed by zinc supplementation. Mini web-site containing index.html and hyperlinked pages in subdirectory describing a Direct Interaction Network automatically generated based on curated interactions contained within the proprietary PathwayArchitect database. Ovals represent proteins and the circles symbolize metal ions. Objects are coloured by their abundance in zebrafish at the time-point they were significantly different from the control is a scale from -4 fold (dark green) to +4 fold (dark red). Where significant differences were found at more than one time-point, the colour overlay shows expression at the first instance. Dark blue squares denote 'binding', and light blue squares 'expression'; green squares stand for 'regulation', green diamonds for 'metabolism', and green circles for 'promoter binding'. Arrow heads indicate directionality of the interaction where annotated. All nodes and edges can be further interrogated by selecting the relative area of the image. [file 1471-2164-11-553-S2.zip › PathwayArchitect Zn xs DIN/688648.html]

# REGULATION:

|  |  |
| --- | --- |
| Type | REGULATION |
| Effect | Negative |


---

|  |  |
| --- | --- |
| Score | 0 |


---

|  |  |
| --- | --- |
| Reference Count | 6 |


---

|  |  |
| --- | --- |
| Mechanism | Unknown |


---

|  |  |
| --- | --- |
| Reference:0 || Sentence | "Respiratory burst activity, an indicator of differentiation, was not affected by TEPA, demonstrating that the reduction of Cu/Zn-SOD activity was due to copper chelation and not due to changes in Cu/Zn-SOD protein levels that occur during differentiation." |
| PMID | 1333521 |
| Year | 1992 |
| Species | Human |
| Journal | J Nutr |
| RefScore | 2 |
| Source | PArchNLP |
  |
|  |  |


---

|  |  |
| --- | --- |
 Reference:1 || Sentence | "Highly elevated copper concentrations in the hair and significantly reduced zinc levels in serum and urine were detected in AGU patients indicating a disturbance of trace element balance." |
| PMID | 3939546 |
| Year | 1985 |
| Species | Human |
| Journal | J Inherit Metab Dis |
| RefScore | 0 |
| Source | PArchNLP |
  ||  |  |


---

|  |  |
| --- | --- |
 Reference:2 || Sentence | "CONCLUSIONS: The inactivation of cytosolic Cu, Zn-SOD by restriction of dietary copper results in the depression of NO-mediated vascular smooth-muscle relaxation probably by interaction of NO with superoxide." |
| PMID | 8714818 |
| Year | 1995 |
| Species | Rat |
| Journal | Microcirculation |
| RefScore | 1 |
| Source | PArchNLP |
  ||  |  |


---

|  |  |
| --- | --- |
 Reference:3 || Sentence | "We observed striking elevation of manganese and, to a lesser extent, of zinc accompanied by significant reduction of copper bound to purified PrP in all sCJD variants, determined by the PrP genotype and PrP(Sc) type, combined." |
| PMID | 11579148 |
| Year | 2001 |
| Species | Human |
| Journal | J Neurochem |
| RefScore | 2 |
| Source | PArchNLP |
  ||  |  |


---

|  |  |
| --- | --- |
 Reference:4 || Sentence | "To evaluate the individual defensive roles of copper, zinc-superoxide dismutase and manganese-super-oxide dismutase, we treated HaCaT cells with diethyldithiocarbamate, a chelating agent of ionic copper that inactivates copper,zinc-superoxide dismutase activities, tumor necrosis factor alpha, which enhances manganese-superoxide dismutase levels, or transforming growth factor beta1, which inhibits manganese-superoxide dismutase levels." |
| PMID | 10692109 |
| Year | 2000 |
| Species | Human |
| Journal | J Invest Dermatol |
| RefScore | 2 |
| Source | PArchNLP |
  ||  |  |


---

|  |  |
| --- | --- |
 Reference:5 || Sentence | Copper (Cu) deficiency decreases the activity of Cu-dependent antioxidant enzymes such as Cu,zinc-superoxide dismutase (Cu,Zn-SOD) and may be associated with increased susceptibility to oxidative stress. |
| Year | 2005 |
| PMID | 16098731 |
| Species | Rat |
| Journal | J Nutr Biochem |
| RefScore | 1 |
| Source | PArchNLP |
  |


---

|  |  |
| --- | --- |
